# Comparison of Biogenic Amines and Mycotoxins in Alfalfa and Red Clover Fodder Depending on Additives

**DOI:** 10.3390/ijerph14040418

**Published:** 2017-04-14

**Authors:** Jiri Skladanka, Vojtech Adam, Ondrej Zitka, Veronika Mlejnkova, Libor Kalhotka, Pavel Horky, Klara Konecna, Lucia Hodulikova, Daniela Knotova, Marie Balabanova, Petr Slama, Petr Skarpa

**Affiliations:** 1 Faculty of AgriSciences, Mendel University in Brno, Zemedelska 1, 61300 Brno, Czech Republic; jiri.skladanka@mendelu.cz (J.S.); vojtech.adam@mendelu.cz (V.A.); ondrej.zitka@mendelu.cz (O.Z.); veronika.mlejnkova@mendelu.cz (V.M.); libor.kalhotka@mendelu.cz (L.K.); pavel.horky@mendelu.cz (P.H.); xhodulik@node.mendelu.cz (L.H.); marie.balabanova@mendelu.cz (M.B.); petr.skarpa@mendelu.cz (P.S.); 2 Research Institute for Fodder Crops, Ltd. Troubsko, Zahradni 1, 66441 Troubsko, Czech Republic; konecna@vupt.cz (K.K.); knotova@vupt.cz (D.K.)

**Keywords:** green matter, silage, enterococci, fungi, butyric acid, tyramine, putrescine, cadaverine, spermine, zearalenone, deoxynivalenol, biological additives, chemical additives

## Abstract

In the production of fermented feed, each crop can be contaminated with a variety of microorganisms that may produce natural pollutants. Biogenic amines, mycotoxins, and undesirable organic acids can decrease health feed safety. The aim of this study was to compare the counts of microorganisms, levels of biogenic amines, and the mycotoxins in forage legumes, and also to compare the occurrence of microorganisms and levels of mycotoxins in green fodder and subsequently produced silage and the influence of additives on the content of natural harmful substances in silage. The experimental plot was located in Troubsko and Vatín, in the Czech Republic. Two varieties of *Medicago sativa* and one variety of *Trifolium pratense* were compared. Green fodder and subsequently produced silage reaching up to 23% of dry matter were evaluated and prepared using a bio-enzymatic additive and a chemical additive. Green fodder of *Medicago sativa* was more contaminated by *Enterococci* than *Trifolium pratense* fodder. The obvious difference was determined by the quality of silage leachate. The silage prepared from *Medicago sativa* fodder was more contaminated with butyric acid. Fungi were present in higher counts in the anaerobic environment of green fodder and contaminated it with zearalenone and deoxynivalenol. Lower counts of fungi were found in silage, although the zearalenone content did not change. Lower content of deoxynivalenol was detected in silage, compared with green fodder. Silages treated with a chemical additive were found not to contain butyric acid. Lower ethanol content was determined, and the tendency to reduce the risk of biogenic amines occurrence was evident. The additives proved to have no influence on the content of mycotoxins.

## 1. Introduction

In the production of fermented feed, each crop is contaminated to a certain extent by a variety of microorganisms (epiphytic microflora). Their effect can be corrected in some way using the silage additives either on a biological basis (e.g., the addition of lactic acid bacteria to the ensiled mass) or the use of chemical additives based on organic acids and their salts [1,2]. Firstly, the additives serve to regulate the fermentation process. The goal of using the additives is to obtain a desirable content of lactic acid (Lac), and through their use it is possible to eliminate the production of undesirable butyric acid (But) produced by *Clostridia*. The basis for biogenic amines (BAs) formation is proteolysis, a naturally occurring process during ensilage comprising the enzymatic decarboxylation of amino acids by the action of plant proteases, peptidases, and the enzymes of various Lac bacteria, clostridia, and other genera [3]. Microorganisms of the genera *Clostridia*, *Bacillus*, *Klebsiella*, *Escherichia*, *Lactobacillus*, and *Pediococcus* can also cause a deep decomposition of protein, resulting in the formation of BAs [4]. The inoculation of *Lactobacillus casei* can lower BA concentrations, while the effects of *Lacobacillus buchneri* may vary considerably. The screening of But-producing activity may help to reduce the risk of contamination in inoculated silage [5]. The application of chemical additives has been shown to reduce Enterobacteriaceae counts and the level of amines in silages [6]. The concentration of BAs in silages mainly depends on the crop during harvest [3]. The risk of BA formation can be present when ensiling protein fodder, such as *Medicago sativa*, especially when appropriately dry matter cannot be provided, and the insufficiently wilted biomass is ensiled [7,8]. The production of roughage is also associated with the risk of mycotoxin occurrence and the possibility of mycotoxins entering the food chain [9]. Mycotoxins are dangerous metabolites that are often carcinogenic, and represent a serious threat to both animal and human health [10]. Mycotoxins are produced by filamentous fungi that can evoke an acute or chronic disease in vertebrate animals in small concentrations [11]. Contamination of food and feed occurs in the field before harvest or during storage, despite the most strenuous efforts of prevention [12]. Deoxynivalenol (DON) and zearalenone (ZEA) are ranked among the most frequently encountered mycotoxins [13]. Trichothecenes, such as deoxynivalenol or nivalenol, are a very large family of chemically related sesquiterpenic mycotoxins produced by various species of *Fusarium* [14]. The aim of this work is (I) to compare the microbial counts and levels of BA and mycotoxins in alfalfa and red clover fodder, (II) to compare the occurrence of microorganisms and the levels of mycotoxins in green fodder and subsequently produced silages, and (III) to assess the effect of additives on the content of natural harmful substances in silages of alfalfa and red clover.

## 2. Materials and Methods

Biomass was assessed from the first cut of alfalfa and red clover. The growths were harvested at the stage of deployment of flower buds (butonization). The experimental plot was located in Troubsko and Vatín, in the Czech Republic. The crop was harvested in June. The species fodder was the first assessed factor was the type of fodder used: *Medicago sativa* L., Holyna variety (1.1), *Medicago sativa* L., Tereza variety (1.2), or *Trifolium pratense* L., Amos variety (1.3). The second evaluated factor was the additive used in the production of silage (2): untreated (2.1), treated with biological additives (2.2), or treated with a chemical additive (2.3). The biological silage additive contained bacteria involved in Lac fermentation, such as *Lactococcus lactis* (NCIMB 30117), *Lactobacillus plantarum* (DSM 16568), and *Enterococcus faecium* (DSM 22502/NCIMB 11181), and enzyme xylanase EC 3.2.1.8 (a hydrolytic enzyme, for increasing the availability of carbohydrates for bacteria). The composition of the chemical silage additive was as follows: 43% formic acid (98%), 30% ammonium formate (98%), 10% propionic acid (99.5%) and 2% benzoic acid. Immediately after harvest, the samples of green fodder were taken, and the occurrence of microorganisms and the content of mycotoxins such as deoxynivalenol (DON) and zearalenone (ZEA) were evaluated on location. The portion of biomass with dry matter content (up to 23%) was preserved by the ensiling process. Representatives of fodder samples (6 kg) were filled into mini-silos using a pneumatic press made of polyvinyl chloride and compacted with a pressure of 600 kg/m. The filled silos (three repetitions per treatment) were sealed with a lid, stored in a room without direct light exposure at room temperature of 28 °C, and stored for 90 days. At the end of the ensiling period (90 days), the silos were opened and samples were taken for the respective chemical analyses. Lac, pH-value, and volatile fatty acids (VFAs) such as acetic acid (Acet), But, and propionic acid (PA) were analyzed in fodder samples 90 days after ensiling (AOAC, 1980). The analytical procedures including the preparation of water extracts have been described previously by Dolezal et al. [1]. Green fodder samples and silages were dried at 60 °C, grounded to a particle size of <1 mm, then analyzed for the content of mycotoxins, such as deoxynivalenol (DON) and zearalenone (ZEA), using the enzyme-linked immunosorbent assay (ELISA, Noack ČR, spol. s r. o., Czech Republic) according to Skladanka et al. [9]. Limits of detection for DON and ZEA were 200–600 ng/mL. PCA agar (Biokar Diagnostics, Paris, France) was used as a culture medium to determine the total number of microorganisms (TNM) and the incubation lasted for 72 h at 30 °C. For the determination of yeasts and fungi, Chloramphenicol Glucose Agar (Biokar Diagnostics, Paris, France) was used, and the incubation lasted 120 h at 25 °C. Lac bacteria (lactic acid bacteria) were determined in MRS (de Man, Rogosa and Sharpe) agar (Biokar Diagnostics, Paris, France) and incubated for 72 h at 30 °C. For the determination of bacteria from the Enterobacteriaceae family, VRBG (Violet Red Bile Glucose) agar (Biokar Diagnostics, Paris, France) was used and the incubation lasted 24 h at 37 °C. For the determination of enterococci, Compass Enterococcus agar (Biokar Diagnostics, Paris, France) was used and the incubation lasted for 24 h at 44 °C. After the incubation in the thermostat, the grown colonies were counted from the Petri dishes, and the results of analyses were expressed as colony forming units (CFU) per gram of green fodder or silage, respectively. AAA 400 (Ingos, Prague, Czech Republic) IEC (Ion Exchange Chromatography) apparatus was used for the evaluation of BA content. The content of histamine (Him), tyramine (Tym), putrescine (Put), cadaverine (Cad), spermidine (Spd), spermine (SPM), and the total content of BA were evaluated. The system consisted of a glass filling chromatographic column and steel pre-column, two chromatographic pumps for transport of elution buffers and derivatization reagent, a cooled carousel for 25 Eppendorf tubes, a dosing valve, a heat reactor, a Vis detector, and a cooled chamber for derivatization reagent. The volume of the injected sample was up to 100 µL with RSD (Relative Standart Deviation) 1%. We used a two-channel Vis detector with a 5 µL flow volume cuvette operated at wavelengths of 440 and 570 nm. A solution of ninhydrin was prepared in 75% methyl cellosolve (v/v) and 25% 4 M acetic buffer (v/v, pH 4.0). Tin chloride was used as a reducing reagent. A prepared solution of ninhydrin was stored under inert atmosphere (N_2_) with cooling at 4 °C. Flow rate was up to 0.25 mL·min^−1^. The pressure ranged from 4.5 to 6.0 MPa. Reactor temperature was set to 120 °C. For elution, two buffers were employed: a buffer was composed of 5.5 mM C_6_H_8_O_7_, 81 mM Na_3_C_6_H_5_O_7_, 257 mM NaCl, 350 mM KBr, and 250 mL of C_3_H_8_O per 1 L of Milli-Q water, with a final pH of 5.78. For pH measurements, the WTW inoLab pH meter (Weilheim, Germany) was employed. The content of ethanol was determined using gas chromatography, as described by Hartman [15].

The data were statistically processed using STATISTICA.CZ, version 10.0 (StatSoft, Prague, Czech Republic). Statistical significance was determined by examining the differences between groups using ANOVA and Scheffé’s test. Box plots of multiple variables were used for graphical representation. Differences with *p* < 0.05 were considered significant. 

## 3. Results

Epiphytic microflora consisted mainly of Lac bacteria (Table 1). A higher proportion of these bacteria was obvious in *Medicago* species, especially of the Tereza variety.

**Figure 1:**
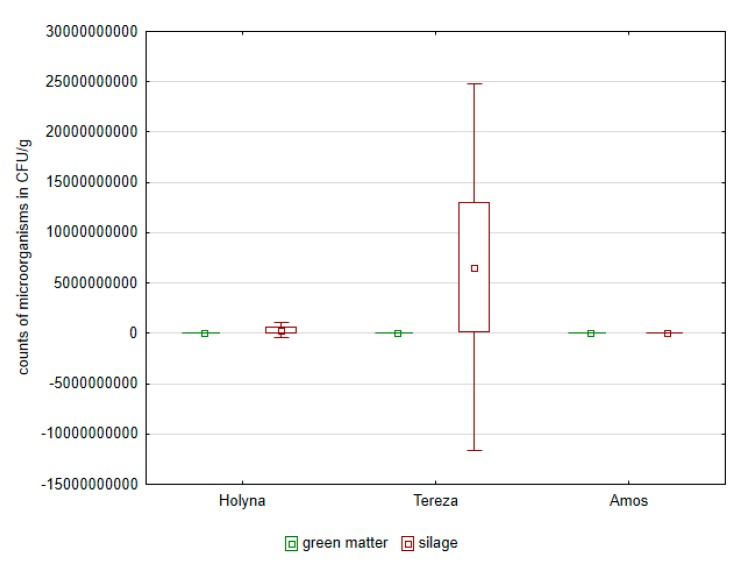
Comparison of Lac bacteria in green mass and in control silages of alfalfa and red clover.

**Figure 2:**
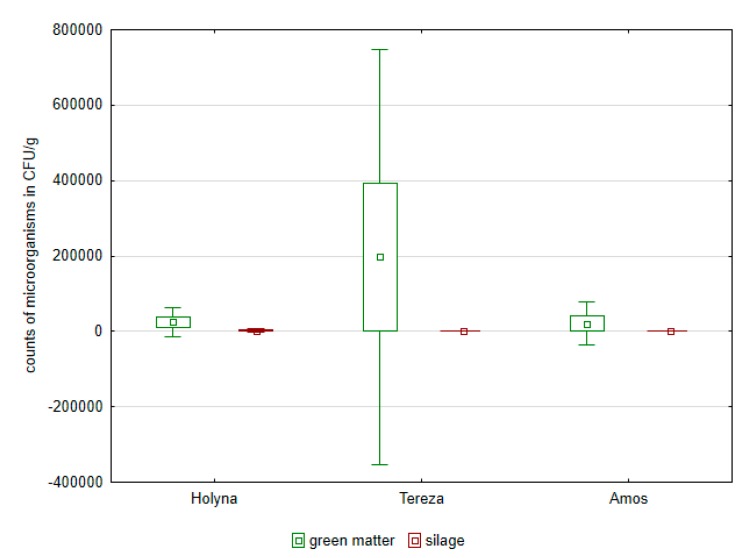
Comparison of Enterobacteriaceae in green mass and in control silages of alfalfa and red clover.

**Figure 3:**
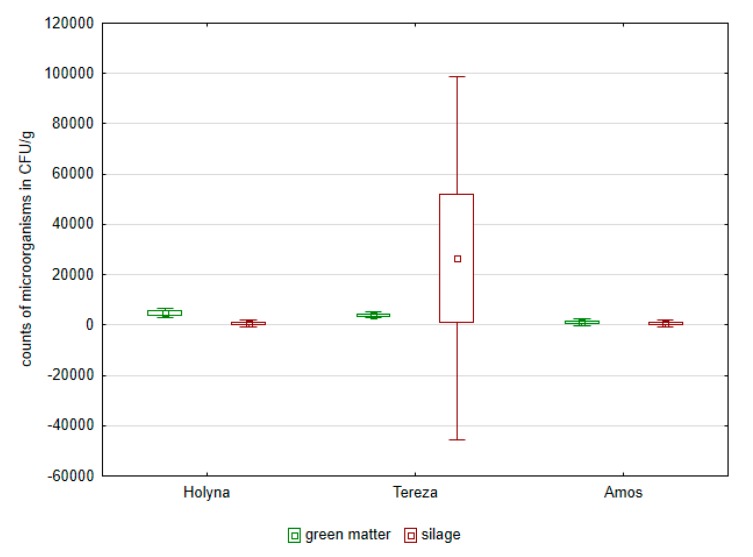
Comparison of enterococci in green mass and in control silages of alfalfa and red clover.

**Figure 4:**
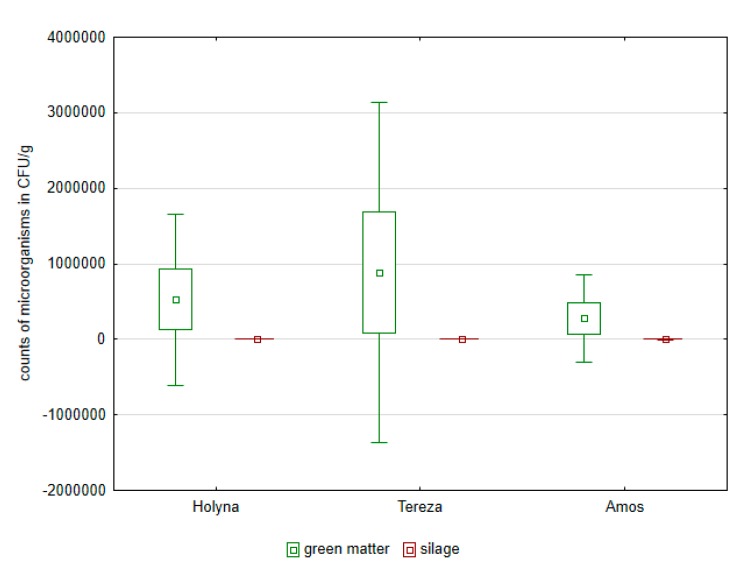
Comparison of fungi in green mass and in control silages of alfalfa and red clover.

Green matter of this variety was more contaminated by bacteria of the Enterobacteriaceae family as well as fungi (Figure 1, Figure 2, Figure 3 and Figure 4). Differences between the species were documented by the occurrence of enterococci, which was higher in *Medicago sativa* than in *Trifolium pratense*. Lac bacteria and enterococci counts were higher in produced silages than in green matter (Figure 1 and Figure 3), which was apparent in *Medicago sativa*, Tereza variety. Conversely, the bacteria proportion of the Enterobacteriaceae family and fungi in produced silages was significantly lower (Figure 2 and Figure 4).

The use of mixtures of organic acids and their salts in the production of silage was reflected in a lower content of TNM, Lac bacteria, bacteria of the Enterobacteriaceae family, fungi, and yeasts. The effect of organic acids and their salts is particularly evident in relation to the occurrence of enterococci. Their counts were lower (*p* < 0.05) in variants with chemical additives, compared to variants with biological additives (Table 2). The difference between the species is also evident with respect to pH and organic acid content in the produced silage. Silage made from *Medicago sativa* proved higher (*p* < 0.05) pH, lower (*p* < 0.05) content of Lac, and higher (*p* < 0.05) content of Acet (Table 3). There were some obvious differences in the occurrence of (But) in silage prepared from *Medicago sativa*, especially in the Tereza variety. A mixture of organic acids, utilized in the preservation, resulted in a decrease (*p* < 0.05) of the Acet content. BA content was decreased (*p* < 0.05) to zero using the organic acids. Between the evaluated species and varieties, respectively, no difference in the content of BA was detected (Table 4). However, in *Medicago sativa*, especially in the Tereza variety, there was an evident trend toward higher total content of BA, histamine, tyramine, and cadaverine.

**Figure 5:**
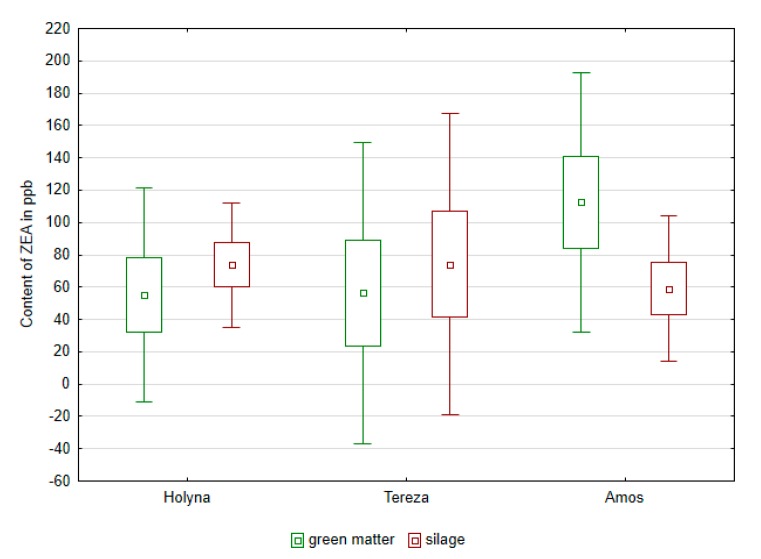
Comparison of the content of zearalenone (ZEA) in green mass and in silages of alfalfa and red clover.

**Figure 6:**
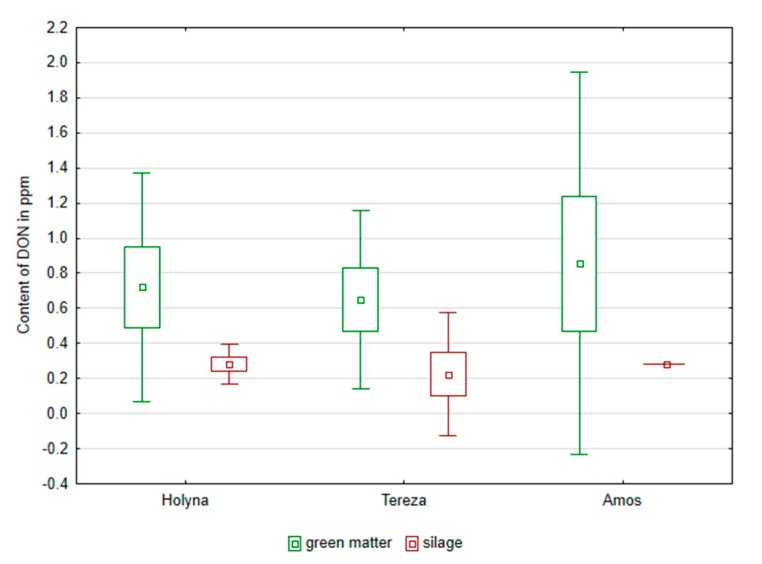
Comparison of the content of deoxynivalenol (DON) in green mass and in silages of alfalfa and red clover silages.

Similarly, it was not possible to confirm the effect of additives on BA content in produced silages, but it was obvious that the use of mixtures of organic acids and their salts in the ensiling production appeared to reduce the total content of BA, tyramine, putrescine, cadaverine, and spermidine, compared to control variant. Green fodder had already been contaminated by mycotoxins before ensilage (Figure 5 and Figure 6). The occurrence of deoxynivalenol (DON) and zearalenone (ZEA) was confirmed. ZEA content ranged from 23.45 to 140.87 ppb in green fodder before silage. The fodder contamination of evaluated species was balanced (the same amongst the samples). Similar ZEA content as those found in green fodder was also found in silage, where values ranged from 41.18 to 107.23 ppb. DON content ranged from 0.47 to 1.24 ppm in green fodder. The fodder contamination of each species was also balanced (the same amongst the samples). In produced silages, the DON content decreased to 0.10 and then up to 0.35 ppb. Significant decrease of DON after treatment with additive was found in experimental variants (Figure 6 and Table 5). A dependence between the content of BA and the content of mycotoxins was not observed (Table 6). An exception was the medium strong (r = 0.61) correlation between the content of putrescine and deoxynivalenol.

## 4. Discussion

Lac bacteria are microorganisms that are necessary for the proper progress of the fermentation. They are facultative anaerobic microorganisms that can produce Lac and Acet. Lac is included in the basic acids used for ensuring the success of the conservation process [16]. These bacteria get into the ensiled biomass while it is still on the field. They were also found in the alfalfa and clover fodder as well. Although, no significant differences were found between the species, it was obvious that *Medicago sativa*, especially the Tereza variety, contained more Lac bacteria, compared with *Trifolium pratense*. An aerobic environment resulted from the low content of green matter, and a higher content of Lac bacteria was associated with anaerobic environment during ensiling. Also in silage, a higher level of Lac bacteria was evident in the Tereza variety. Similarly, enterococci are Lac bacteria, but their determination relates to their ability to produce BA. These bacteria occur in biological silage additives [17]. The use of biological additive is reflected in an increase (*p* < 0.05) of their counts, compared with the control variant and the variant treated with the mixtures of organic acids and their salts. This increase is not reflected in the content of BA. The use of biological additive was not shown to affect the content of BA. This finding contrasted with the results of Nishino et al. [5], who stated that biological additives containing *Lactobacillus casei* can reduce the content of BA in silage. However, these results were obtained in silage of festulolia and maize. Simultaneously, Van Os et al. [18] confirmed the fact that unless the acidification of silage was set in the first 10 days of fermentation, the production of BA would be significantly increased. Mainly the content of cadaverine in silages treated with chemical additive was decreased. Although, this reduction was not significant, the observed tendency confirmed the fact that the chemical additives were more suitable for preservation of biomass with low dry matter content in comparison to biological additives, with regard not only to the quality of the ensiling process, but also the content of toxic products. Moreover, the use of chemical additive resulted in a decrease (*p* < 0.05) in pH of the silage to a value of 4.22. The pH value influenced the activity of the decarboxylases [4] and also inhibited the growth of microorganisms [19]. Low (*p* < 0.05) pH value was also observed in silage produced from biomass of *Trifiolium pratense* L. The species was detected with lower counts of TNM, Lac bacteria, Enterobacteriaceae, and especially enterococci. In the evaluated silages, tyramine, putrescine, and spermine were mainly detected. Putrescine is considered to be one of the factors causing ketosis [20]. Tyramine increases blood pressure very effectively. Spermine affects the growth and division of cells. Tyramine, putrescine, and cadaverine reduce the palatability of silage for cattle. Histamine is among the most harmful amines. It induces a reduction in blood pressure and worsens blood circulation in the extremities of cattle [7]. Our results indicated low histamine content in the evaluated silages. The highest content was found out in *Medicago sativa*, especially in the Tereza variety (16.5 mg·kg^−1^). Steidlova and Kalac [21] detected histamine only in grass silage not treated with additives (7.4 mg·kg^−1^). Silages treated with chemical and biological additives were devoid of histamine. In contrast, our results showed the occurrence of histamine in alfalfa and red clover silage not only in untreated samples, but also in silages treated with chemical and biological additive. This could be the result of a low content of dry matter of ensiling biomass. Nishino et al. [5] listed the content of histamine up to 195 mg·kg^−1^ in untreated grass silage. On the other hand, they mentioned that silage treated with biological additives reduced its content only by 10.3 to 18.9 mg·kg^−1^. Higher effectiveness of microorganisms stimulates amine degradation and thus prevents amine accumulation in the rumen. In practice, content of amines may be relevant, especially in short term during the change of feed, since amines are rapidly degraded in adapted animals. Histamine was degraded to the greatest extent followed by tyramine, putrescine, and cadaverine [3]. Feeding silage spiked with histamine in increments up to 1 g·day^−1^ to sheep over a period of seven days [22] and 5 g·day^−1^ to heifers had no impact on dry matter intake [23]. Holzer et al. [24] stated that undesirable microflora consisting of bacteria (including enterobacteria and clostridia), yeasts, and fungi involved in the impairment of feed could consequently cause health complications to animals or changes in animal products. Bacteria of the Enterobacteriaceae family are microorganisms capable of producing the undesirable But. High counts of Enterobacteriaceae were detected in green biomass of *Medicago sativa*, especially in the Tereza variety. The occurrence of these microorganisms in green fodder can greatly affect the success of the silage process. This fact corresponded with high (*p* < 0.05) levels of BA in *Medicago sativa* silages. The Enterobacteriaceae counts in silages treated with organic acids and their salts were apparently lower, although with no significant difference. The effect of chemical additives was confirmed by Selwet et al. [6] reporting that the use of a chemical additive called KemiSile 2000 (Mikrop Čebín a.s., Čebín, Czech Republic) can reduce the occurrence of Enterobacteriaceae, in particular in silage wilted to 20% of dry matter. Herrmann et al. [25] confirmed the inhibition of undesirable but alcohol and ammonia formation after the application of chemical additives. Fungi were particularly present in *Medicago sativa*. A significant difference in the contents of DON and ZEA was not detected between the evaluated species. Fungi are aerobic microorganisms that are present during harvesting [26] and the ensilage process in the anaerobic environment reduces their counts. Particularly, it can be evident in the use of chemical additives. Currently, no acceptable limits exist for counts of fungi and yeasts in the Czech Republic. Fungi in alfalfa and red clover silage were detected in the order of magnitude 10^2^–10^3^ CFU·g^−1^. Scudamore and Livesey [27] reported that the occurrence of fungi in counts higher than 10^4^ CFU·g^−1^ can induce animal health problems. Alonso et al. [28] stated that the occurrence of fungi is related to the lack of hygienic quality of silage leading to loss of nutrients and dry matter. Mycotoxins entered silage directly in the field. A difference in the content of mycotoxins between green fodder and silage was not observed. In the case of DON, a reduction of mycotoxin was apparent.

## 5. Conclusions

Green fodder of *Medicago sativa* was found to be more contaminated with microorganisms than *Trifolium pratense* fodder. A higher content of Lactobacillus, bacteria of the Enterobacteriaceae family, fungi, and yeasts was evident, but significant difference was found only in the counts of enterococci. Despite the differences in the counts of enterococci, a difference in the content of BA was not detected between the evaluated subsequently produced silages. Indeed, the counts of microorganisms were found to be comparable in the silage mass. In the evaluated species, the content of mycotoxins was not found to be different. A difference was observed in the quality of silage leachate. Silages prepared from *Medicago sativa* fodder were more contaminated with But. Green fodder was characterized by low counts of Lac bacteria and enterococci, while the Lac bacteria and enterococci were recorded in higher numbers in produced silages. In contrast, bacteria of the Enterobacteriaceae family and fungi were determined to be in higher quantities in the aerobic environment of green fodder. Although lower counts of fungi were detected, zearalenone content remained the same. In comparison with the green fodder, the content of deoxynivalenol was lower in silages. The contamination of mycotoxins was shown to originate from the field. The zearalenone content remained the same in the produced silage. On the other hand, the content of deoxynivalenol was reduced by the ensiling process. The content of natural pollutants was mainly influenced by a mixture of organic acids. Silages treated with a chemical additive displayed lower content of ethanol. Tendency to reduce the risk of BA content was obvious. The additives had no influence on the content of mycotoxins. Based on the results, it can be stated that chemical additives (mixture of organic acids) are convenient treatments to be used within the ensiling process of the insufficiently wilted fodder of *Medicago sativa* and *Trifolium pratense*.

## Figures and Tables

**Table 1:** Total number of microorganisms (TNM), lactobacilli, enterobacteria, enterococci, yeasts, and fungi in colony forming units CFU·g^−1^ in alfalfa and red clover green mass.

Species	TNM	Lac Bacteria	Enterobacteria	Enterococcus	Fungi	Yeasts
*Medicago sativa* (Holyna)	6.625 × 10^7^	1.315 × 10^5^	2.500 × 10^4^	4.795 × 10^3 a^	5.250 × 10^5^	5.500 × 10^3^
*Medicago sativa* (Tereza)	1002.515 × 10^7^	3.930 × 10^5^	19.725 × 10^4^	4.070 × 10^3 a,b^	8.822 × 10^5^	9.500 × 10^3^
*Trifolium pratense* (Amos)	3.834 × 10^7^	0.525 × 10^5^	2.100 × 10^4^	1.000 × 10^3 b^	2.760 × 10^5^	2.275 × 10^3^
*p* value	0.4649	0.4430	0.5306	0.0330	0.7412	0.3482

Values with different lowercase letters indicate significant differences at *p* < 0.05.

**Table 2:** Total number of microorganisms (TNM), Lac bacteria, enterobacteria, enterococci, yeasts, and fungi in CFU·g^−1^ in alfalfa and red clover silages.

Factor	TNM	Lac Bacteria	Enterobacteria	Enterococcus	Fungi	Yeasts
Species (S)						
*Medicago sativa* (Holyna)	9.783 × 10^9^	6.027 × 10^8^	1.127 × 10^3^	1.714 × 10^4^	1.863 × 10^3^	4.037 × 10^4^
*Medicago sativa* (Tereza)	16.336 × 10^9^	11.804 × 10^8^	0.562 × 10^3^	2.152 × 10^4^	0.354 × 10^3^	0.0016 × 10^4^
*Trifolium pratense* (Amos)	1.418 × 10^9^	0.237 × 10^8^	0.234 × 10^3^	1.725 × 10^4^	3.123 × 10^3^	4.145 × 10^4^
*p* value	0.5545	0.4495	0.4526	0.9386	0.1491	0.4746
Additives (P)						
Control	22.096 × 10^9^	16.063 × 10^8^	0.422 × 10^3^	0.509 × 10^4 a^	1663	5.886 × 10^4^
Biological additive	5.211 × 10^9^	1.829 × 10^8^	1.395 × 10^3^	5.015 × 10^4 b^	2938	2.041 × 10^4^
Chemical additive	0.231 × 10^9^	0.176 × 10^8^	0.106 × 10^3^	0.673 × 10^4 a^	741	0.257 × 10^4^
*p* value	0.2602	0.1722	0.1832	0.0023	0.2907	0.3356
S × P	0.5136	0.5935	0.9747	0.9915	0.6926	0.7375

Values with different lowercase letters indicate significant differences at *p* < 0.05.

**Table 3:** Content of Lac, Acet, propionic acid (PA), biogenic amines (BAs), ethanol (%), and the pH value in alfalfa and red clover silages.

Factor	pH	Lac	Acet	PA	BA	Ethanol
Species (S)						
*Medicago sativa* (Holyna)	4.64 ^a^	7.33 ^a^	3.43 ^a^	0.032	1.056 ^a,b^	0.995 ^a^
*Medicago sativa* (Tereza)	4.81 ^a^	6.25 ^a^	3.52 ^a^	0.058	1.279 ^a^	1.275 ^b^
*Trifolium pratense* (Amos)	4.14 ^b^	11.25 ^b^	2.59 ^b^	0.000	0.000 ^b^	1.193 ^a,b^
*p* value	0.0000	0.0000	0.0041	0.0731	0.0205	0.0301
Additives (P)						
Control	4.82 ^a^	9.03	3.59 ^a^	0.000 ^a^	1.056 ^a,b^	1.555 ^a^
Biological additive	4.54 ^a^	7.99	4.07 ^a^	0.091 ^b^	1.279 ^a^	1.350 ^a^
Chemical additive	4.22 ^b^	7.81	1.88 ^b^	0.000 ^a^	0.000 ^b^	0.557 ^b^
*p* value	0.0000	0.4344	0.0000	0.0004	0.0003	0.0000
S × P	0.0002	0.4679	0.0001	0.0369	0.0292	0.0465

Values with different lowercase letters indicate significant differences at *p* < 0.05.

**Table 4:** Content of histamine (Him), tyramine (Tym), putrescine (Put), cadaverine (Cad), spermidine (Spd), spermine (SPM), and the total content of BA (mg·kg^−1^ of dry matter) in alfalfa and red clover silages.

Factor	Him	Tym	Put	Cad	Spd	Spm	BA
Species (S)							
*Medicago sativa* (Holyna)	4.5	233.5	241.0	21.7	2.50	219.5	722.7
*Medicago sativa* (Tereza)	16.5	459.8	211.0	71.5	1.83	204.8	965.8
*Trifolium pratense* (Amos)	9.0	73.0	356.8	50.8	3.00	225.5	872.2
*p* value	0.2873	0.1513	0.7087	0.3857	0.6857	0.8686	0.8045
Additives (P)							
Control	7.5	321.8	410.5	80.2	2.83	197.7	1174.8
Biological additive	12.5	420.3	282.7	63.7	3.00	225.8	1008.2
Chemical additive	9.0	24.2	115.7	0.17	1.50	226.3	377.7
*p* value	0.7882	0.1251	0.3145	0.0992	0.4885	0.7200	0.1269
S × P	0.5617	0.6775	0.4641	0.7238	0.4930	0.5826	0.5572

**Table 5:** Content of deoxynivalenol (DON) (ppm) and zearalenone (ZEA) (ppb) in alfalfa and red clover silages.

Factor	DON	ZEA
Species (S)		
*Medicago sativa* (Holyna)	0.4517	115.35
*Medicago sativa* (Tereza)	0.3883	125.08
*Trifolium pratense* (Amos)	0.4983	115.55
*p* value	0.7198	0.7861
Additives (P)		
Control	0.7417 ^a^	76.66 ^a^
Biological additive	0.2933 ^b^	144.04 ^b^
Chemical additive	0.3033 ^b^	137.28 ^b^
*p* value	0.0129	0.0031
S × P	0.9620	0.0953

Average values in the columns with superscripts a,b are significant at *p* < 0.05.

**Table 6:** Correlation between the content of BA and mycotoxins.

BA	DON	ZEA
Him	−0.14	0.30
Tym	−0.17	−0.29
Put	0.61	0.12
Cad	0.18	−0.25
Spd	0.18	0.13
Spm	−0.12	−0.06
BA	0.43	−0.08
